# Reviewed article: Kale PL, Alt NN, Fonseca SC. Prevalence of congenital abnormalities among live-born children: a cross-sectional study in the state of Rio de Janeiro, Brazil, 2019-2021. Epidemiol Serv Saude. 2025:34;e20240471.

**DOI:** 10.1590/S2237-96222025v34e2024471.a

**Published:** 2025-05-12

**Authors:** Sílvia Helena Mendonça de Moraes

**Affiliations:** Fiocruz Mato Grosso do Sul

**Table:** 

Rating	Excellent	Good	Average	Below average	Poor
Interest		✓			
Quality			✓		
Originality			✓		
Overall			✓		

**Table:** 

Questionnaire	Yes	No	Not applicable
Does the manuscript contain new and significant information to justify publication?	✓		
Does the Abstract (Summary) clearly and accurately describe the content of the article?		✓	
Is the problem significant and concisely stated?		✓	
Are the methods described comprehensively?		✓	
Are the interpretations and conclusions justified by the results?	✓		
Is adequate reference made to other work in the field?	✓		

## Manuscript Structure:

Length of article is: Adequate

Number of tables is: Adequate

Number of figures is: Too little

Please state any conflict(s) of interest that you have in relation to the review of this paper (state “none” if this is not applicable): There is no conflict of interest.

## Comments

1) Não há claramente uma pergunta de pesquisa. Como o delineamento do estudo não está muito claro, sugiro incluir a pergunta de pesquisa e explicar com mais detalhes como o delineamento do estudo ajudará a responder à pergunta.

2) Descreva os métodos com mais detalhes, sobretudo o processo de coleta e anonimização de dados (incluindo o acesso ao SINASC), para que outros pesquisadores possam replicar o estudo. Explique como os vieses foram minimizados e a validade interna foi garantida. Os métodos precisam ser descritos com mais detalhes para permitir a replicação do estudo por outros pesquisadores.

3) As tabelas precisam ser revisadas quanto à língua portuguesa. A indicação de fonte precisa ser inserida ao final de cada tabela.

4) No Resumo, melhore a redação do objetivo e conclusão que o responda a partir dos resultados apresentados, pois, a redação da conclusão colocada no Resumo parece que não “conversa” com o objetivo do estudo.

5) O texto precisa ser revisado e ampliado em algumas partes, como, por exemplo: o Contexto (explicar o que significa o Rio de Janeiro ser o oitavo no Indice de Desenvolvimento Social e em que isso se relaciona (ou implica) ao (no) seu estudo) e o Tamanho do estudo (explicar melhor o que está considerando como “elegível” e “não elegível”).

6) O manuscrito precisa ser revisado para corrigir erros gramaticais e ortográficos.

